# Disease Waves of SARS-CoV-2 in Iran Closely Mirror Global Pandemic Trends

**DOI:** 10.34172/aim.2022.83

**Published:** 2022-08-01

**Authors:** Zohreh Fattahi, Marzieh Mohseni, Maryam Beheshtian, Ali Jafarpour, Khadijeh Jalalvand, Fatemeh Keshavarzi, Hanieh Behravan, Fatemeh Ghodratpour, Farzane Zare Ashrafi, Marzieh Kalhor, Maryam Azad, Mahdieh Koshki, Azam Ghaziasadi, Mohamad Soveyzi, Alireza Abdollahi, Seyed Jalal Kiani, Angila Ataei-Pirkooh, Iman Rezaeiazhar, Farah Bokharaei-Salim, Mohammad Reza Haghshenas, Farhang Babamahmoodi, Zakiye Mokhames, Alireza Soleimani, Zohreh Elahi, Masood Ziaee, Davod Javanmard, Shokouh Ghafari, Akram Ezani, Alireza Ansari Moghaddam, Fariba Shahraki-Sanavi, Seyed Mohammad Hashemi Shahri, Azarakhsh Azaran, Farid Yousefi, Afagh Moattari, Mohsen Moghadami, Hamed Fakhim, Behrooz Ataei, Elahe Nasri, Vahdat Poortahmasebi, Mojtaba Varshochi, Ali Mojtahedi, Farid Jalilian, Mohammad Khazeni, Abdolvahab Moradi, Alijan Tabarraei, Ahmad Piroozmand, Yousef Yahyapour, Masoumeh Bayani, Fatemeh Tavangar, Mahmood Yaghoubi, Fariba Keramat, Mahsa Tavakoli, Tahmineh Jalali, Mohammad Hassan Pouriayevali, Mostafa Salehi-Vaziri, Hamid Reza Khorram Khorshid, Reza Najafipour, Reza Malekzadeh, Kimia Kahrizi, Seyed Mohammad Jazayeri, Hossein Najmabadi

**Affiliations:** ^1^Genetics Research Center, University of Social Welfare and Rehabilitation Sciences, Tehran, Iran; ^2^Kariminejad-Najmabadi Pathology & Genetics Center, Tehran, Iran; ^3^Research Center for Clinical Virology, Tehran University of Medical Sciences, Tehran, Iran; ^4^Gerash Amir-al-Momenin Medical and Educational Center, Gerash University of Medical Sciences, Gerash, Iran; ^5^Department of Pathology, School of Medicine, Imam Khomeini Hospital, Tehran University of Medical Sciences, Tehran, Iran; ^6^Department of Virology, School of Medicine, Iran University of Medical Sciences, Tehran, Iran; ^7^Department of Medical Microbiology, Antimicrobial Resistance Research Center, Communicable Diseases Institute, Faculty of Medicine, Mazandaran University of Medical Sciences, Sari, Iran; ^8^Department of Molecular Diagnostic, Emam Ali Educational and Therapeutic Center, Alborz University of Medical Sciences, Karaj, Iran; ^9^Department of Infectious Diseases, Imam Ali hospital, Alborz University of Medical Sciences, Karaj, Iran; ^10^Infectious Diseases Research Center, Birjand University of Medical Sciences, Birjand, Iran; ^11^Qazvin Deputy of Treatment Reference Laboratory, Qazvin University of Medical Sciences, Qazvin, Iran; ^12^Health Promotion Research Center, Zahedan University of Medical Science, Zahedan, Iran; ^13^Infection Disease and Tropical Medicine Research Center, Zahedan University of Medical Science, Zahedan, Iran; ^14^Department of Medical Virology, School of Medicine, Ahvaz Jundishapur University of Medical Sciences, Ahvaz, Iran; ^15^Infectious and Tropical Diseases Research Center, Health Research Institute, Ahvaz Jundishapur University of Medical Sciences, Ahvaz, Iran; ^16^Department of Virology, School of Medicine, Shiraz University of Medical Sciences, Shiraz, Iran; ^17^Health Policy Research Center, Shiraz University of Medical Sciences, Shiraz, Iran; ^18^Infectious Diseases and Tropical Medicine Research Center, Isfahan University of Medical Sciences, Isfahan, Iran; ^19^Department of Bacteriology and Virology, Faculty of Medicine, Tabriz University of Medical Sciences, Tabriz, Iran; ^20^Infectious and Tropical Disease Research Center, Tabriz University of Medical Science, Tabriz, Iran; ^21^Microbiology Department, School of Medicine, Guilan University of Medical Sciences, Rasht, Iran; ^22^Department of Medical Virology, Faculty of Medicine, Hamadan University of Medical sciences, Hamadan, Iran; ^23^Booali Lab, Molecular & Virology Diagnostic Section, Qom, Iran; ^24^Golestan University of Medical Sciences, Gorgan, Iran; ^25^Department of Microbiology, School Of Medicine, Kashan University of Medical Sciences, Kashan, Iran; ^26^Infectious Diseases and Tropical Medicine Research Center, Babol University of Medical Sciences, Babol, Iran; ^27^Iranian Blood Transfusion Research Center, High Institute for Education and Research in Transfusion Medicine, Tehran, Iran; ^28^Aramesh Pathology & Genetics laboratory, Tehran, Iran; ^29^Brucellosis Research Center, Hamedan University of Medical Science, Hamadan, Iran; ^30^COVID-19 National Reference Laboratory, Pasteur Institute of Iran, Tehran, Iran; ^31^Department of Arboviruses and Viral Hemorrhagic Fevers (National Reference Laboratory), Pasteur Institute of Iran, Tehran, Iran; ^32^Digestive Disease Research Institute, Shariati Hospital, Tehran University of Medical Sciences, Tehran, Iran

**Keywords:** COVID-19, Iran, SARS-CoV-2, Whole genome sequencing

## Abstract

**Background:**

Complete SARS-CoV-2 genome sequencing in the early phase of the outbreak in Iran showed two independent viral entries. Subsequently, as part of a genome surveillance project, we aimed to characterize the genetic diversity of SARS-CoV-2 in Iran over one year after emerging.

**Methods:**

We provided 319 SARS-CoV-2 whole-genome sequences used to monitor circulating lineages in March 2020-May 2021 time interval.

**Results:**

The temporal dynamics of major SARS-CoV-2 clades/lineages circulating in Iran is comparable to the global perspective and represent the 19A clade (B.4) dominating the first disease wave, followed by 20A (B.1.36), 20B (B.1.1.413), 20I (B.1.1.7), leading the second, third and fourth waves, respectively. We observed a mixture of circulating B.1.36, B.1.1.413, B.1.1.7 lineages in winter 2021, paralleled in a fading manner for B.1.36/B.1.1.413 and a growing rise for B.1.1.7, prompting the fourth outbreak. Entry of the Delta variant, leading to the fifth disease wave in summer 2021, was detected in April 2021. This study highlights three lineages as hallmarks of the SARS-CoV-2 outbreak in Iran; B4, dominating early periods of the epidemic, B.1.1.413 (B.1.1 with the combination of [D138Y-S477N-D614G] spike mutations) as a characterizing lineage in Iran, and the co-occurrence of [I100T-L699I] spike mutations in half of B.1.1.7 sequences mediating the fourth peak. It also designates the renowned combination of G and GR clades’ mutations as the top recurrent mutations.

**Conclusion:**

In brief, we provided a real-time and comprehensive picture of the SARS-CoV-2 genetic diversity in Iran and shed light on the SARS-CoV-2 transmission and circulation on the regional scale.

## Introduction

 Since December 2019, when the severe acute respiratory syndrome coronavirus 2 (SARS-CoV-2) was detected in Wuhan,^1^ real-time whole-genome sequencing began scrutinizing its genome. Such genome surveillance projects aim to measure transmission, monitor viral changes over time, and recognize the emerging viral variants.^2^ Simultaneously, broad and dynamic systems categorized the circulating SARS-CoV-2 variants, such as GISAID and Nextstrain nomenclature systems for clade assignment and Pangolin, a more dynamic algorithm for lineage classification.^3-5^ So far, multiple lineages/sub-lineages have emerged from the two major A and B lineages at the phylogeny root, and now 1293 Pango lineages, 12 Nextstrain and, 9 GISAID clades are circulating worldwide.^6^ These variants may arise out of one or a combination of deviant mutations,^7^ and the variants with higher transmissibility and severe disease waves are known as variants of concern (VOC), including Alpha (20I clade, B.1.1.7 lineage),^8^ Beta (20H clade, B.1.351 lineage),^9^ Gamma (20J clade, P.1 lineage),^10^ and Delta (21A clade, B.1.617.2 lineage).^11^

 During the COVID-19 pandemic, different studies have monitored circulating SARS-CoV2 lineages on global and regional scales. In December 2020, Cella et al studied 261 487 public genome sequences and reported B and B.1 as the most prevalent lineages in the world.^12^ Such studies are also routinely performed on regional scales. For instance, analysis of 353 SARS-CoV-2 genomes in South-Eastern Italy introduced 20E, 20B, and 20A as the circulating clades in over one year with the dominancy of 20E.^13^ During November 2020-March 2021, another study in the USA reported 20G, 20I, 20C, 20A, and 20B as the most predominant circulating clades by large scale sequencing of SARS-CoV2 genomes.^14^

 Iran is one of the most affected Asian countries that confronted the spread of SARS-CoV-2 in at least five significant infection waves as of September 2021. The first COVID-19 patients were officially announced in February 2020, which soon led to the first outbreak peak in March, continuing Mid-May 2020. At that point, whole-genome sequencing of 50 viral isolates from the early phase of the epidemic revealed B4 (19A clade) as the main SARS-CoV-2 lineage while detecting a surge of B.1.* lineages from May 2020 onward.^15^ The country faced a second increase in coronavirus patients/deaths from early June to the end of August 2020, and then the third wave gradually started in early September to the end of December 2020. Fortunately, a relative decrease in the number of patients was declared for the entire country from January 2021 to the end of March. However, insufficient restrictions during the national holidays in March caused a notable rise in the number of patients. Eventually, the fourth outbreak peak started from April 2021 until early June, and the highest death rate from the beginning of the pandemic was recorded on April 27, 2021 (496 daily deaths). In less than two weeks, the fifth outbreak peak started from the southern parts of Iran and expanded to the northern regions, reaching 709 daily deaths on August 25, 2021.

 The current study investigates complete genome sequences of SARS-CoV-2 viral isolates from Iran in the time interval of March 2020-May 2021. We aimed to characterize SARS-CoV-2 genetic diversity and circulating lineages/clades, especially those corresponding to the four infection waves of the disease. To accomplish this, we started with genome sequencing of 50 SARS-CoV-2 samples from the early phase of the epidemic^15^ and then continued sequencing of additional 319 viral isolates to provide a comprehensive picture of the SARS-CoV-2 epidemic in Iran, over one year after emerging.

## Materials and Methods

###  Specimen Recruitment, Sequencing, and Genome Assembly

 As part of a genome surveillance program, 319 viral RNAs extracted from the respiratory tract samples of laboratory-confirmed patients were collected from October 2020 till the end of May 2021 (Table S1). Most samples were collected randomly from different cities in Iran with the help of the Iranian Network for Research in Viral Diseases (INRVD) or from private laboratories. However, a minor fraction of viral samples suspected of a special VOC were obtained through contact tracing and screening programs conducted in the Pasteur Institute of Iran (IPI). Whole-genome sequencing was performed following the CleanPlex® SARS-CoV-2 Research and Surveillance Panel (Paragon Genomics, Inc.) protocol. The targeted libraries were then paired-end sequenced on Illumina MiSeq instrument using 300-cycle MiSeq v2 reagent kits (Illumina, Inc.). On average, 98.8% of the SARS-CoV-2 reference genome (NC_045512.2) was covered with an average depth of 1886.55X.

 Initially, FASTQ files were assessed by FastQC,^16^ then processed and compared with two in-house pipelines simultaneously. FASTQ pre-processing was performed with Fastp^17^ and Cutadapt^18^ programs, followed by alignment to the NC_045512.2 using Bowtie2^19^ or Burrows-Wheeler Aligner,^20^ keeping the high-quality reads mapped in proper pair. Then, consensus SARS-CoV-2 sequences were assembled using Samtools mpileup and Bcftools.^21^ Finally, the consensus FASTQ files were converted into FASTA format by Seqtk (https://github.com/lh3/seqtk), masking bases with quality lower than 20 to ambiguous nucleotides (N). Furthermore, the automated Genome Detective system was also applied in parallel for the low-quality samples.^22^

###  Lineage and Clade Assignment, Mutation Analysis

 Linage and clade assignment was performed by Pangolin v3.1.7^5^ and NextClade v.1.5.2^3^ for a total of 369 sequences obtained by adding the original 50 FASTA files generated from the initial phase of this study.^15^ As the samples were recruited in two separate projects, sampling in July-September 2020 interval was incomplete. Virus tracking in those months was accomplished using additional 14 samples submitted by other groups in GISAID (Table S2). Next, 25 sequences containing > 5% ambiguous nucleotides (N) and bad overall NextClade QC score were removed from the cohort of samples subjected for mutation analysis. Detecting substitutions, deletions, and insertions were performed relative to NC_045512.2, using NextClade and Coronavirus Typing Tool.^23^

## Results

###  Cohort Description

 In total, 369 SARS-CoV-2 samples were ascertained from patients with a history of COVID-19 symptoms and positive real-time RT-PCR. These samples were obtained from 19 different provinces during a time interval starting from March 2, 2020 until May 27,2021 (Figure 1). The patient’s age ranged from 11 to 93 years (mean: 45.35 years.); 201 (54.5%) men (mean: 44.38 years.), 168 women (mean: 46.50 years.). A total of 136 (36.9%) samples were taken from hospitalized patients, while 207 (56.1%) individuals were outpatients at the time of sample collection (Table S1). This cohort comprises at least ten samples monthly and covers nearly the four peaks of SARS-CoV-2 spread in Iran; 188 (51%) of the samples were obtained during the outbreak peaks, and the other half were collected to track the viral changes in time intervals flanked by the outbreak peaks.

**Figure 1:**
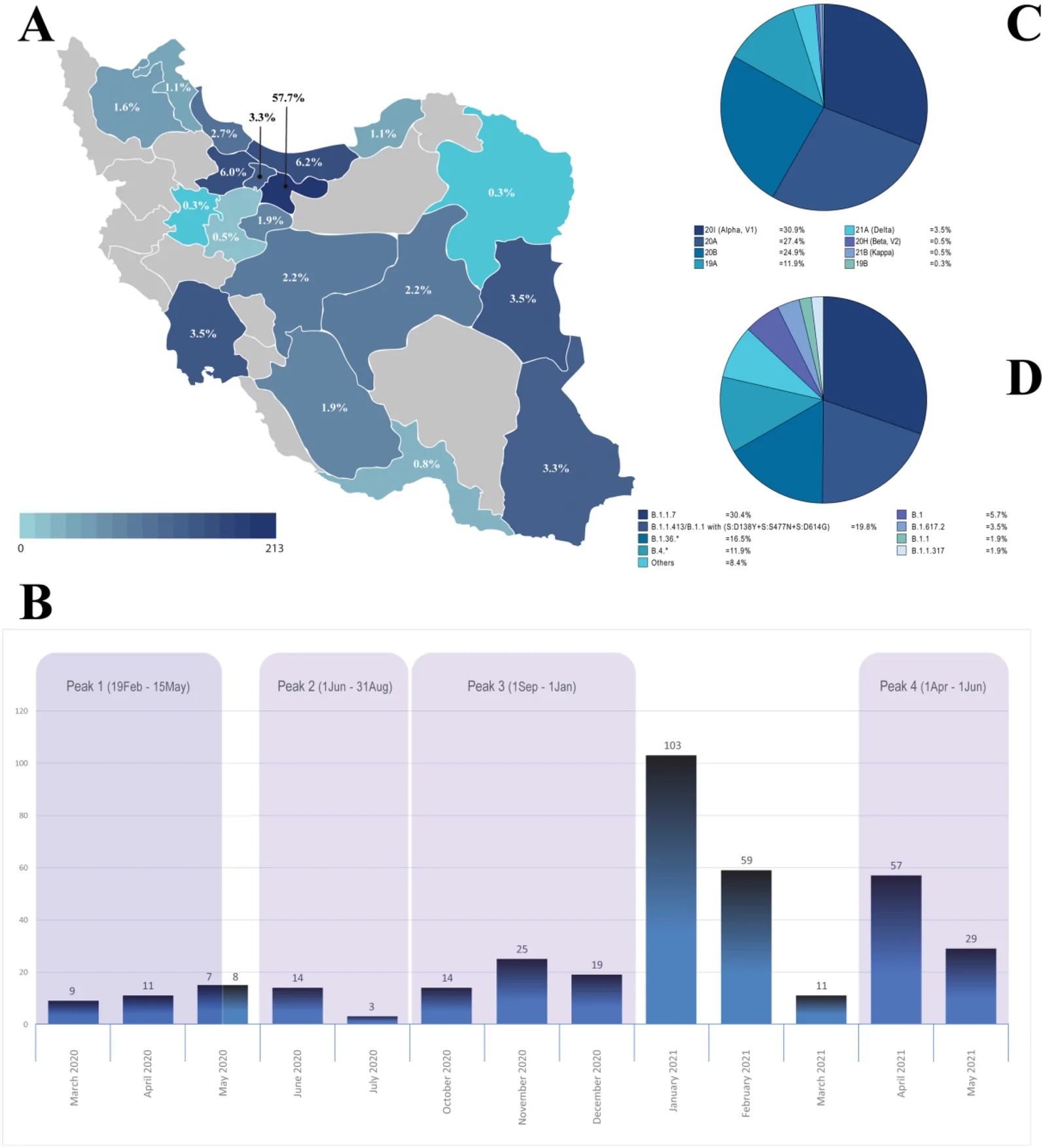
Distribution of SARS-CoV-2 Genome Sequences in This Study; Geographical **(A)** and chronological **(B)** and major circulating SARS-CoV-2 clades **(C)** and lineages **(D)** in Iran

 The following time intervals were considered for the four disease waves as follow; First peak (February 19 –May 15,2020), Second peak; summer 2020 (June 1 –August 31, 2020), Third peak; autumn 2020 (September 1, 2020 –January 1, 2021), Fourth peak; spring 2021 (April 1 – June 2021) (https://covid19.who.int/region/emro/country/ir). The number and percent of samples taken from each city, each month, and each outbreak peak are shown in Figures 1A and 1B. The observed inflation in the number of samples ascertained in January 2021 [103 (27.9%)] is due to the need for tracking the possible entry of the Alpha variant into the country at that critical time.

###  Major Circulating SARS-CoV-2 Lineages/Clades in Iran

 Over one year of the SARS-CoV-2 epidemic, we detected at least 35 different lineages and eight clades circulating in the country. The most frequent clades are 20I (Alpha, V1), 20A, 20B, and 19A, with the frequency of 31%, 27%, 25%, and 12%, respectively (Figure 1C). The 35 lineages comprise ~2.7% of Pango lineages circulating worldwide,^6^ with B.1.1.7, B.1.1.413/B.1.1 carrying (S:D138Y + S:S477N + S:D614G), B.1.36.* and B.4.* being the most frequent lineages observed, with 30%, 20%, 16.5%, and 12% frequency, respectively (Figure 1D). We claim that all viral isolates of our cohort except one sample with “A23.1 lineage” belong to the “B lineage”, which mirrors the epidemic status in Iran during this time interval. Remarkably, no rise based on the entry of the “A lineage” was observed in the country.

###  Temporal Investigation of SARS-CoV-2 Epidemic in Iran

####  First Wave of SARS-CoV-2 Infection (February 19-May 15, 2020)

 The epidemic in Iran started with 19A clade/B4 lineage (See Figure 2, Figure 3, and Figure S1 for a detailed overview), imported from China, which dominated the first outbreak peak and existed significantly until the end of June 2020.^15^ The top missense mutations commonly dominating the first outbreak peak were nsp6:L37F and nsp2:V198I, while only constituting 14% and 11% of the total, respectively (Figure 4A, Figure S2). The frequency of these two mutations decreased gradually; as in August 2020, they were not among the top 20% mutations and then completely disappeared from the epidemic along with the B4 lineage, comparable to the decaying global tendency reported for L37F. The first and prominent spike mutation was T22I (13%), which was replaced upon the entry of the D614G mutation in May 2020. Overall, the T22I frequency in our cohort is considerably low (2%) and disappeared from July 2020 onwards. However, this mutation is still circulating globally as of August 2021.^24^

**Figure 2:**
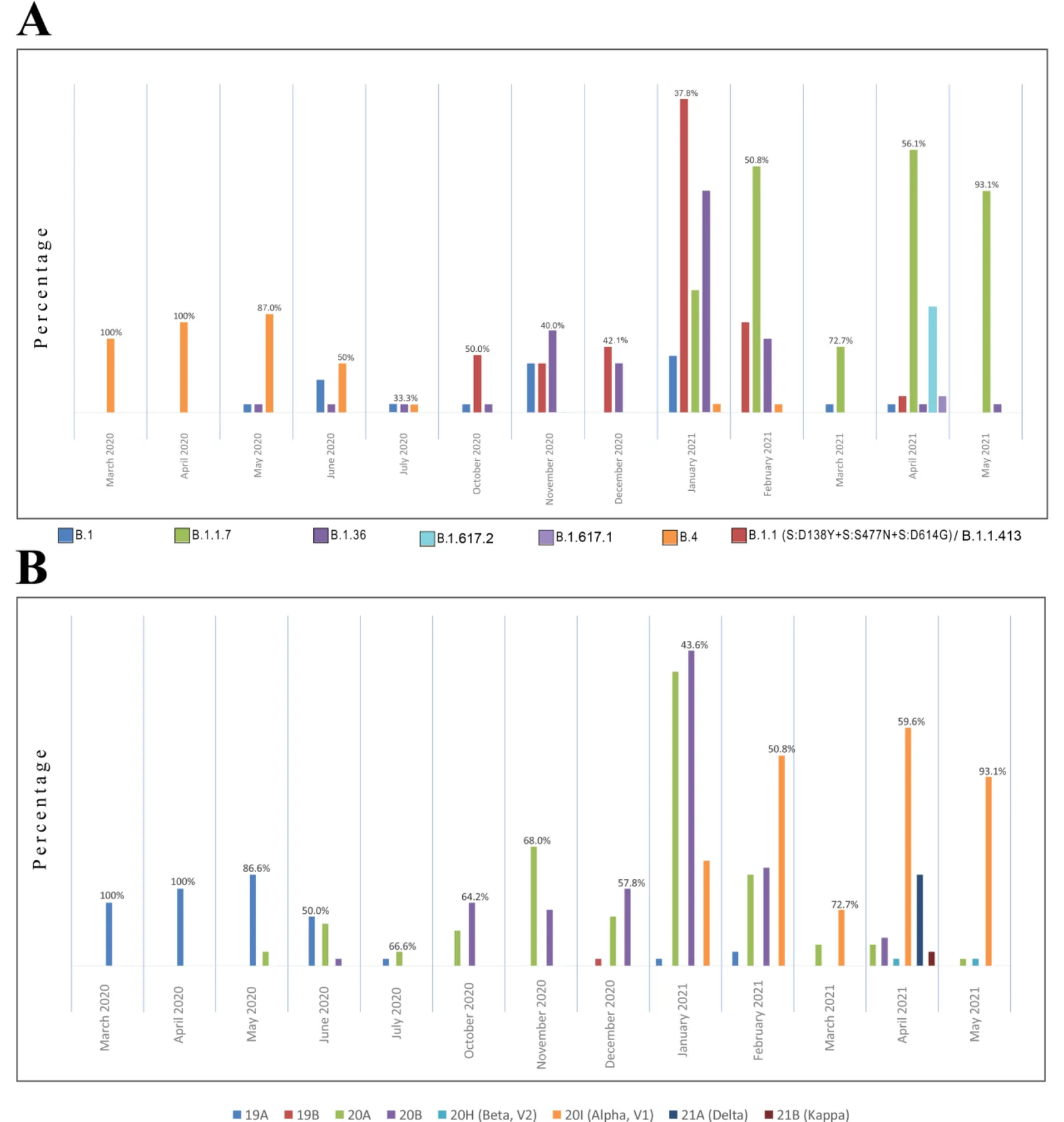
Monthly Investigation of Prominent Lineages **(A)** and Clades **(B)** of SARS-CoV-2 Epidemic in Iran

**Figure 3:**
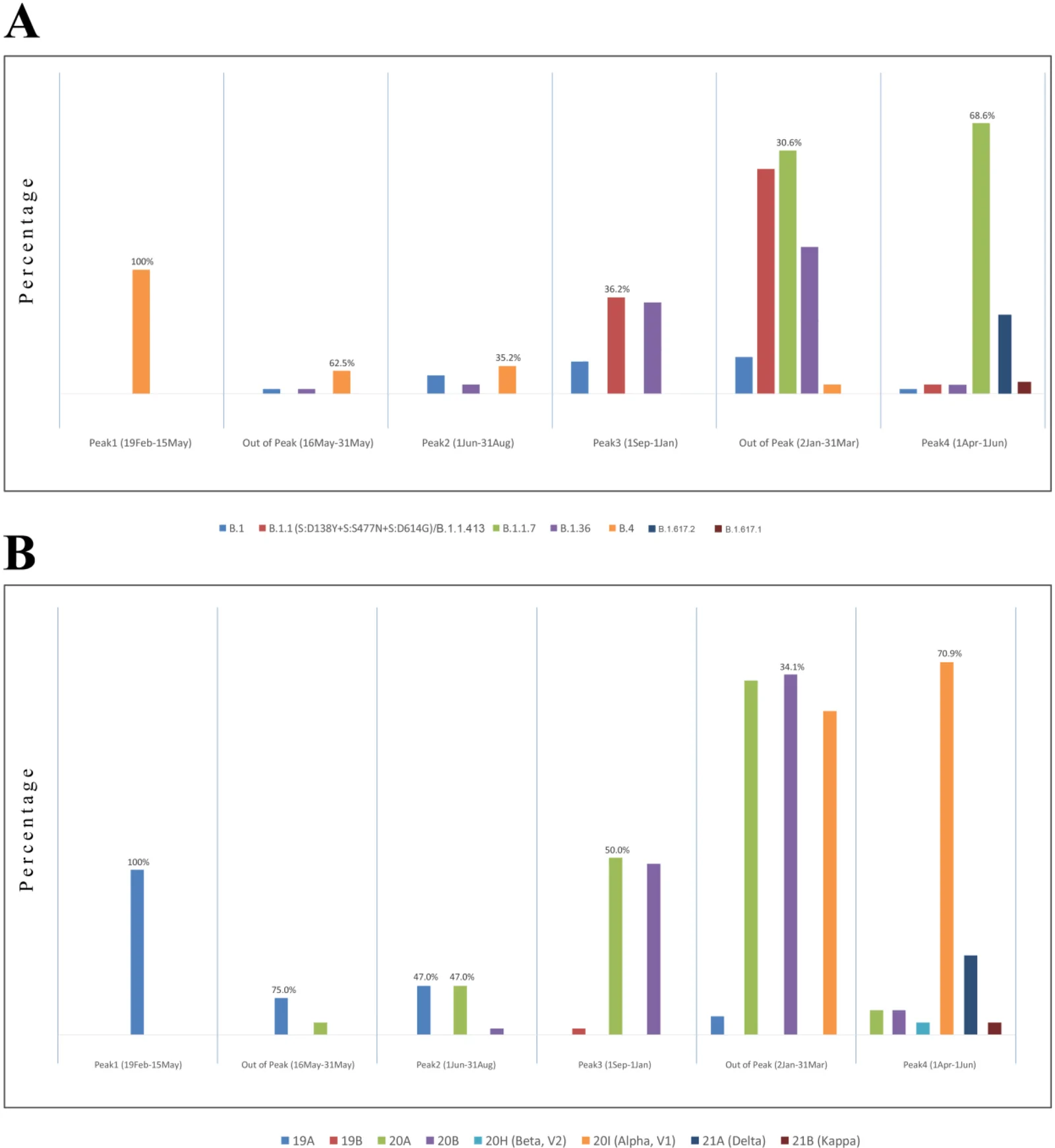
Prominent Lineages **(A)** and Clades **(B)** Based on the Disease Waves of SARS-CoV-2 Epidemic in Iran

**Figure 4:**
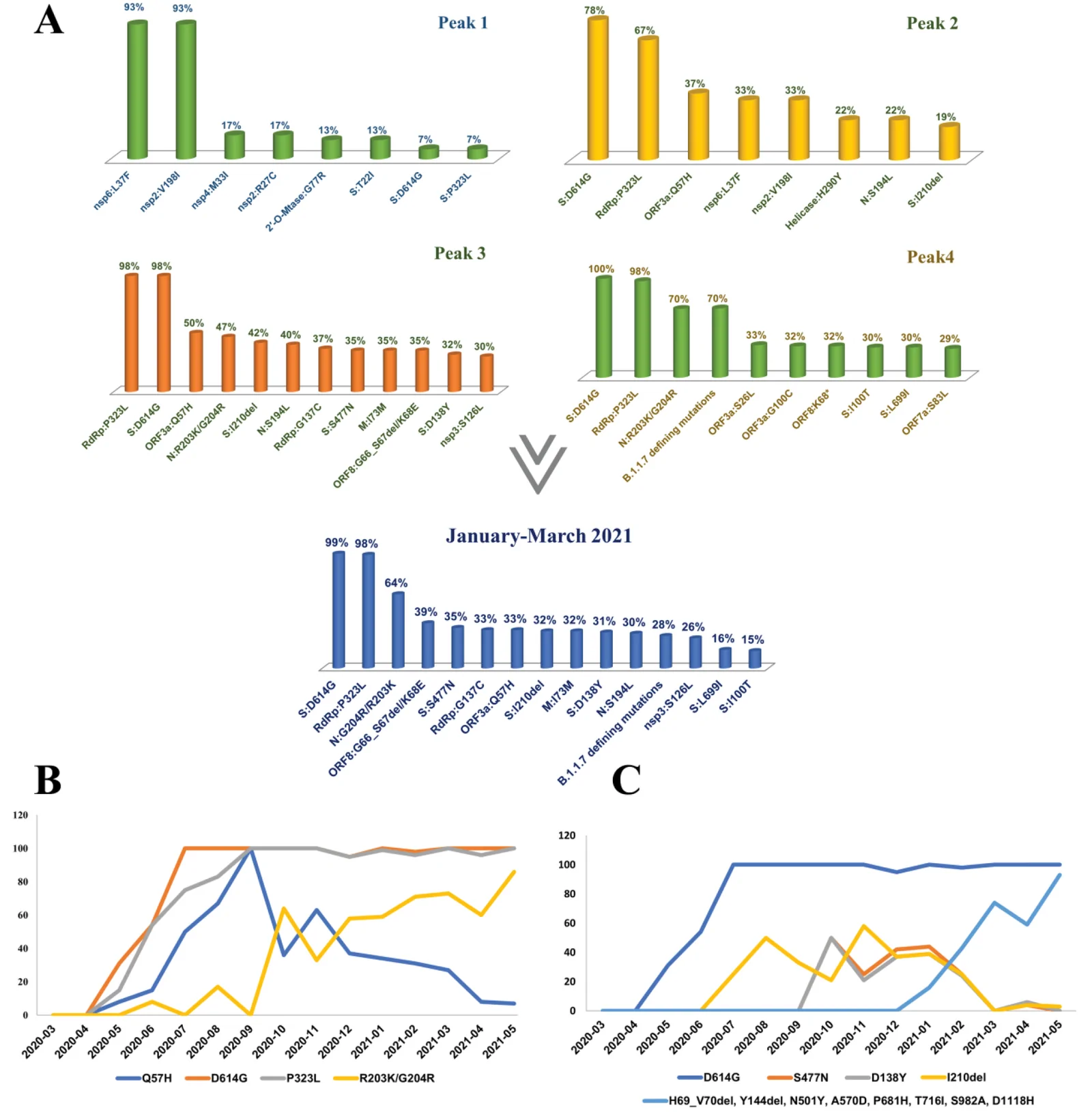
Top Mutations Based on the Disease Waves of SARS-CoV-2 Epidemic in Iran **(A)**. Chronological trend of top lineage-defining **(B)** and Spike **(C)** mutations

 Later, the well-known S:D614G and RdRp:P323L mutations started to appear in Iran from the end of May 2020 and gradually increased in the following months, raising the second disease wave (Figure S2).

####  Second Wave of SARS-CoV-2 Infection; Summer 2020 (June 1–August 31, 2020)

 In May 2020, the 20A clade was observed in our cohort for the first time, represented by B.1 and B.1.36 lineages. The B.1.36 was more frequent and showed an increasing ratio from June to the end of February 2021. Due to the insufficiency of sampling during this peak, the 14 samples submitted by other groups in GISAID in the July-September interval were also investigated, which confirmed this pattern (not shown in Figure 2, see Table S2). Therefore, the 20A clade (led by the B.1.36 lineage) can be considered one of the causes of the second disease wave in summer 2020, possibly along with B.4 (Figure 3).

 In accordance with the above finding, the top missense mutations commonly dominating the second outbreak peak were S:D614G, RdRp:P323L, and ORF3a:Q57H, constituting 91%, 88%, and 28% of the total, respectively (Figure 4A). This new mutational tendency correlated with the entry of B.1* lineages and dominancy of 20A clade in the second peak and is comparable to the world as the D614G mutation gradually dominated the pandemic while mostly accompanying P323L mutation.^25^ The next prominent mutation in this peak was ORF3a:Q57H, first detected in May 2020, which gradually increased in the second disease wave, also persisted in the third peak (50%), and then disappeared from the top 20% mutations of the fourth peak (Figure 4B, Figures S2-S6). Furthermore, as the B4 lineage was still circulating in the country, we could still observe the nsp6:L37F and nsp2:V198I mutations (33%), but with significantly decreased frequency compared to the first peak (Figure 4A, Figure S3)

 The prominent spike mutation other than D614G in this peak was I210del, first observed in July 2020, and holding a similar mutational tendency as Q57H and S194L (Figure 4C). This deletion was mainly present in B.1.36* sequences (73%) in Iran. However, its co-occurrence with S:D138Y, S:S477N and B.1.1.7 sequences was observed but never led to a significant rise in frequency.

####  Third Wave of SARS-CoV-2 Infection; Autumn 2020 (1 September 2020–1 January 2021)

 In June 2020, the first 20B clade was detected (B.1.1.317: Russian lineage), but the clade became frequent only from October 2020, led by a distinct B.1.1 lineage carrying the D138Y, S477N, and D614G spike mutations. The prominent co-occurrence of these spike mutations was the characterizing point of this wave of infection. This lineage constitutes ~80% of the 20B clades in Iran, prompting the third disease wave in autumn 2020 in cooperation with the 20A clade (B.1.36) (See Figure 2, Figure 3, and Figure S1 for a detailed overview).

 At that time, the specific mutational combination of [D138Y-S477N-D614G] in the spike could be detected in sequences of different lineages around the world, such as Brazil (some assigned as B.1.128), the UK, USA, and Russia (designated as B.1.1.317 with S:D138Y + S:S477N + S:A845S).^26^ Still, the corresponding sequences from Iran were assigned as a general B.1.1 lineage by Pangolin. Eventually, with the increas number of similar sequences in GISAID and mainly from this cohort, ~80% of the sequences were assigned as a distinct lineage named “B.1.1.413” by pangolin V.3.1.11 (2021-08-24). This distinct lineage carries the following characterizing mutations [ORF1a:S944L (nsp3: S126L), ORF1b:G128C (RdRp:G137C), ORF1b:P314L (RdRp:P323L), S:D138Y, S:S477N, S:D614G, M:I73M, ORF8:G66E, ORF8:del67/68, N:R203K/G204R]^27^ and was significantly prominent in the SARS-CoV-2 epidemic in Iran. However, B.1.1.413 was initially detected on September 7, 2020 in the UK (EPI_ISL_566603), and at least four other similar sequences from Australia and Canada were available in GISAID before its first detection in Iran (24 October 2020), proposing the possible import of this lineage rather than being formed in the country. Still, we cannot accurately determine its earliest date in Iran because of a gap in the sequences pertaining to August-September 2020. Overall, we introduce B.1.1.413 as a characterizing lineage in Iran during the autumn-winter 2020 infection wave.

 The top missense mutations commonly dominating the third outbreak peak were S:D614G, RdRp:P323L, N:R203K/G204R, and ORF3a:Q57H, while constituting 91%, 88%, 53%, and 28% of the total mutations, respectively (Figure 4A, Figure S4). This new mutational tendency correlated with the prominence of 20B clade, led by B.1.1.413. Besides, RdRp:G137C and M:I73M mutations also showed high frequency in this peak, as characteristic mutations of the B.1.1.413 lineage.

 The prominent spike mutations other than D614G in the third disease wave were I210del, S477N, and D138Y.

 D138Y is one of the defining mutations of P.1 lineage and is located in the N-terminal domain (NTD) of the S1 protein. This missense mutation accompanying other P.1 defining mutations in the NTD can reduce neutralization of mAb159 by disrupting its epitope.^28^ However, D138Y mutation in our cohort did not accompany other P.1 mutations, but co-occurred with S477N and D614G. Although this combination itself is remarkable, as S477N, is well-known to increase viral affinity for ACE2 receptor, and escape multiple mAbs,^29,30^ we cannot justify whether D138Y accompanying other spike mutations (S477N-D614G) still shows the reduction in mAb159 neutralization. The current study shows that S477N was first detected in October 2020 in Iran and was one of the frequent spike mutations not only in autumn 2020 but in winter 2021 (Figure 4C). Later, the S477N and D138Y were seen accompanying some B.1.1.7 sequences but never showed a significant rise.

####  Forth Wave of SARS-CoV-2 Infection; Spring 2021 (April 1–June 2021)

 The 20A and 20B clades dominated the epidemic until the end of January 2021, when the first 20I (Alpha, V1) clade/B.1.1.7 lineage was detected in Tehran. Subsequently, the increase in 20I could be observed until May, constituting 93% of the sequences and mediating the fourth disease wave in spring 2021. However, as shown in Figure 2, 20A and 20B did not entirely disappear and could still be detected in random samples but with a diminishing ratio until May. In this study, the time interval between the official third and fourth peaks (January-March 2021) was carefully investigated, aiming to track the entry and accumulation of B.1.1.7 VOC, as well as chasing the fate of circulating lineages in autumn 2020. The B.1.1.7 was first detected in September but notified as an outbreak in December 2020 in the UK. Similarly, after its first detection in January 2021 in Iran, the surge in hospitalization and deaths was officially observed three months later in April, although in some southern provinces, such as Khuzestan, the disease wave started sooner in winter 2021.

 The top missense mutations commonly dominating this time interval were S:D614G, RdRp:P323L, and N:R203K/G204R, the same as the last peak. However, the frequency of Q57H started to drop, while we see an increasing slope for N:R203K/G204R (Figure 4B). This mutational trend correlates with the increase in 20I and the fading trend of the 20A and 20B clades from January to March 2021. Therefore, based on the approximate parallel frequency of these clades in this interval, the prominent spike mutations other than D614G were S477N, I1210del, D138Y, and the defining B.1.1.7 spike mutations; H69_V70del, Y144del, N501Y, A570D, P681H, T716I, S982A, and D1118H (Figure 4A, Figure S5).

 Subsequently, the seventeen B.1.1.7 defining mutations were observed with 70% frequency in the fourth outbreak peak (Figure 4A, Figure S6), with two new spike mutations, I100T and L699I, in 30% of sequences. Indeed, half of the B.1.1.7 sequences (46.5%) carried these two mutations, while the other half (53.5%) carried the defining mutations uniquely or accompanying other random spike mutations. This combination gave a low global frequency of 0.5%, while Iran showed the most cumulative prevalence.^31,32^ Therefore, the involvement of these two mutations in B.1.1.7 sequences is a characteristic feature of the B.1.1.7 epidemic in Iran. However, their contribution to the epidemic dropped by a third compared to the typical B.1.1.7 sequences (Figure S6).

 The surge of the Delta variant/B.1.617.2 VOC (24%) in April pertained to non-random samples and did not show a high frequency of this variant at that time point.This justifies the lower ratio of B.1.1.7 mutations in this month compared to March (Figures S5 and S6) and confirms the entry of this VOC into the country. The G142D spike mutation was present in 75% of B.1.617.2 sequences (Figure S6), which is not a specific observation, as G142D accompanies 64% of B.1617.2 sequences worldwide.^33^

###  Diversity and Distribution of Variants of Concern and Variants of Interest (VOIs) in SARS-CoV-2 epidemic in Iran

 Overall, we detected the Alpha, Beta, and Delta variants in our cohort. The Alpha variant (B.1.1.7) comprised 31% of our cohort corresponding to the fourth disease wave. The first B.1.1.7 sequence was detected in January 2021 by a Tehrani passenger. In the upcoming months, tracking the viral isolates showed fast-growing replication, starting from 14.6% of samples in January, exceeding 50% of samples in March, and reaching almost 93% of random samples in May.

 The Beta variant (B.1.351) was detected in two non-random samples from the south (Hormozgan province) and southeast of Iran (Sistan and Baluchestan province) in April-May 2021 but did not cause a separate outbreak peak.

 The Delta variant (B.1.617.2) was first detected in non-random samples from the Yazd and Qom provinces collected in April 2021 and then in a random sample in the same month, alarming that this variant was already dispersed in the capital, which eventually led to the fifth disease wave in summer 2021. The dominancy of the Delta variant in this peak is confirmed by Sanger sequencing screening of the spike region of random samples from Tehran (unpublished data). The kappa variant (B.1.617.1) was the only variants of interest detected in non-random samples from the Alborz province in April, and there is a possibility that this variant accompanied the Delta variant in the formation of the fifth outbreak peak.

 Furthermore, A.23.1 (variant of note) was detected once in our cohort; a sub-lineage of A.23, dominating the Uganda epidemic in March 2021^34^ and the only A lineage (19B clade) observed in the SARS-CoV-2 epidemic in Iran. The A.23.1 is an international lineage carrying F157L, V367F, Q613H, and P681R mutations of potential biological concern. This lineage was detected in a random sample from Tehran in December 2020 but no longer in the upcoming months.

###  Mutation Surveillance of SARS-CoV-2 Epidemic in Iran

 In this cohort, 1577 distinct nucleotide mutations were identified in total, of which 853 (54%) disturb the SARS-CoV-2 proteome, including 825 (96.6%) missense, 22 (2.6%) deletion and 6 (0.7%) stop gain/loss mutations. Of the 1577 distinct nucleotide mutations, 1068 (67.7%) were observed only once in the sequences (1050 substitutions and 18 deletions). Similar to other studies, missense mutations comprised the largest percentage of nucleotide mutations (52.5%).^35^ Monthly analysis of the SARS-CoV-2 proteome variations showed an average of 20 mutations per sequence, with two different tendencies ranging from 14.9 before the entry of B.1.1.7 lineage, rising into 34.4 from January 2021, which is rational as this lineage introduces 23 new mutations into the SARS-CoV-2 genome.^5^

 The ten top frequent mutations, regardless of the sequence collection dates, are shown in Table 1. Among these, six mutations represent > 50% of the sequences, including four missense mutations (D614G, P323L, R203K, and G204R), one synonymous (3037C > T; F105F), and one non-coding variant (241C > T). Thus, 88.6% (317/358) of the sequences carry the quadruplet of [A23403G-C14408T-C241T-C3037T], which are the most frequent mutations worldwide,^36^ corresponding to the G clade.^37^ This quadruplet of mutations co-occurs with R203K/G204R in 53% (188/358) of sequences, the two subsequent top mutations defining the GR clade.^37^ Furthermore, the Q57H mutation, the third top missense mutation in the USA,^36^ is observed with 28% (99/358) frequency.

**Table 1:** The 10 Top Frequent Mutations in SARS-CoV-2 Epidemic in Iran

**Mutation**	**Genome Segment**	**Mutation Type**	**Frequency in Iran**
D614G (23403A > G)	Spike (S)	Missense	91%
241C > T	5′-UTR noncoding region	Non-coding	88%
F105F (3037C > T)	Nsp3	Synonymous	88%
P323L (14408C > T)	RNA-dependent RNA polymerase (RdRp)	Missense	88%
R203K (28881G > A28882G > A)	Nucleocapsid (N)	Missense	53%
G204R (28883G > C)	Nucleocapsid (N)	Missense	53%
28271delA	Upstream of the Nucleocapsid (N)	Non-coding	34%
S235F (28977C > T)	Nucleocapsid (N)	Missense	32%
Q27*(27972C > T)	ORF8	Stop gain	31%
Y144del (21992_21994delTAT)	Spike (S)	Deletion	31%

 The remainder carry [G1397A-T28688C-G29742T] mutations of the B.4 lineage (11%), which while dominant ( > 70%) at the early phase,^15^ were replaced by [C241T-C3037T-C14408T-A23403G] after more than one year from the start of the epidemic. Moreover, the most frequent missense mutation at the early phase was Nsp2:V198I, replaced by the renowned D614G mutation (91%). D614G was first detected on May 1, 2020 in Iran and not typically along with [C241T-C3037T-C14408T] but with the defining mutations of the B.4 lineage [G1397A-T28688C-G29742T]. Later in the same month (May 18, 2020), we could detect this mutation accompanying [C241T-C3037T-C14408T], which also carries the second top missense mutation in our cohort: P323L.

 Subsequently, we investigated our cohort for the fifteen most frequent missense variants ( > 5%) in the world, reported by Miao et al. As shown in Table 2, we only shared the top four missense variants. Although Q57H, S194L, S477N, and L37F mutations are present with high frequencies in Iran, they are not among the top 15. A222V, V30L, A220V, T85I, L18F, S24L, I120F have no or considerably low frequency and occur typically in the 20C, 20E, 20F, and 20G clades which are absent from the SARS-CoV-2 epidemic in Iran (Table 2). On the other hand, the subsequent top 11 missense mutations in Iran are mostly the defining B.1.1.7 mutations, mainly caused by the different time intervals of the two cohorts rather than the distinct genetic diversity of SARS-CoV-2 in Iran compared to the world. Miao et alinvestigated 260 673 sequences between December 2019-January 12, 2021, before the dominance of the B.1.1.7 lineage in the world.^38^

**Table 2:** Investigation of the World 15 Top Mutations (Until January 2021) to SARS-CoV-2 Epidemic in Iran

**Variants**	**SARS-CoV-2 Proteome**	**Frequency in World**	**Frequency in Iran**	**Iran/World**	**Main Substitutions Characterizing the SARS-CoV-2 Clades**
D614G	Spike	93.88%	91%	Top 1/ Top 1	20A, 20A.EU2, 20B, 20C, 20E (EU1), 20H, 20I
P323L	NSP12	93.74%	88%	Top 2/ Top 2	20A, 20A.EU2, 20B, 20C, 20E (EU1), 20H, 20I
R203K	Nucleocapsid	28.45%	53%	Top 3/ Top 3	20B, 20I/501Y.V1
G204R	Nucleocapsid	28.13%	53%	Top 4/ Top 4	20B, 20I/501Y.V1
A222V	Spike	26.14%	2%	Not among the 15 Top variants/ Top5	20E (EU1)
V30L	ORF10	26.01%	Not detected	Not among the 15 top variants / Top6	20E (EU1)
A220V	Nucleocapsid	25.96%	2%	Not among the 15 top variants / Top7	20E (EU1)
Q57H	ORF3a	23.60%	28%	Not among the 15 top variants / Top8	20C, 20H/501Y.V2
T85I	NSP2	15.38%	0.6%	Not among the 15 top variants / Top9	20C, 20H/501Y.V2
L18F	Spike	12.08%	0.3%	Not among the 15 top variants / Top10	NA
S194L	Nucleocapsid	6.29%	22%	Not among the 15 top variants / Top11	NA
S477N	Spike	6.62%	22%	Not among the 15 top variants / Top12	20A.EU2, 20F
L37F	NSP6	6.52%	14%	Not among the 15 top variants / Top13	20G
S24L	ORF8	5.24%	Not detected	Not among the 15 top variants / Top14	20G
I120F	NSP2	5.23%	Not detected	Not among the 15 top variants / Top15	20F

###  Mutational Profile of SARS-CoV-2 Viral Components in Iran

 The 31 proteins encoded by 14 open reading frames (ORFs) in the SARS-CoV-2 genome are arranged into 16 non-structural proteins (Nsps; encoded by ORF1ab gene region), four structural proteins (Spike; S, Envelope; E, Membrane; M, and Nucleocapsid; N) and 11 accessory proteins (ORF3a, ORF3b, ORF3c, ORF3d, ORF6, ORF7a, ORF7b, ORF8, ORF9b, ORF9c, and ORF10).^39^ SARS-CoV-2 proteins show different mutation rates correlating with their structural and functional features, and the most recurrent mutation of each protein in our cohort is shown in Figure 5A.^25,35^

**Figure 5:**
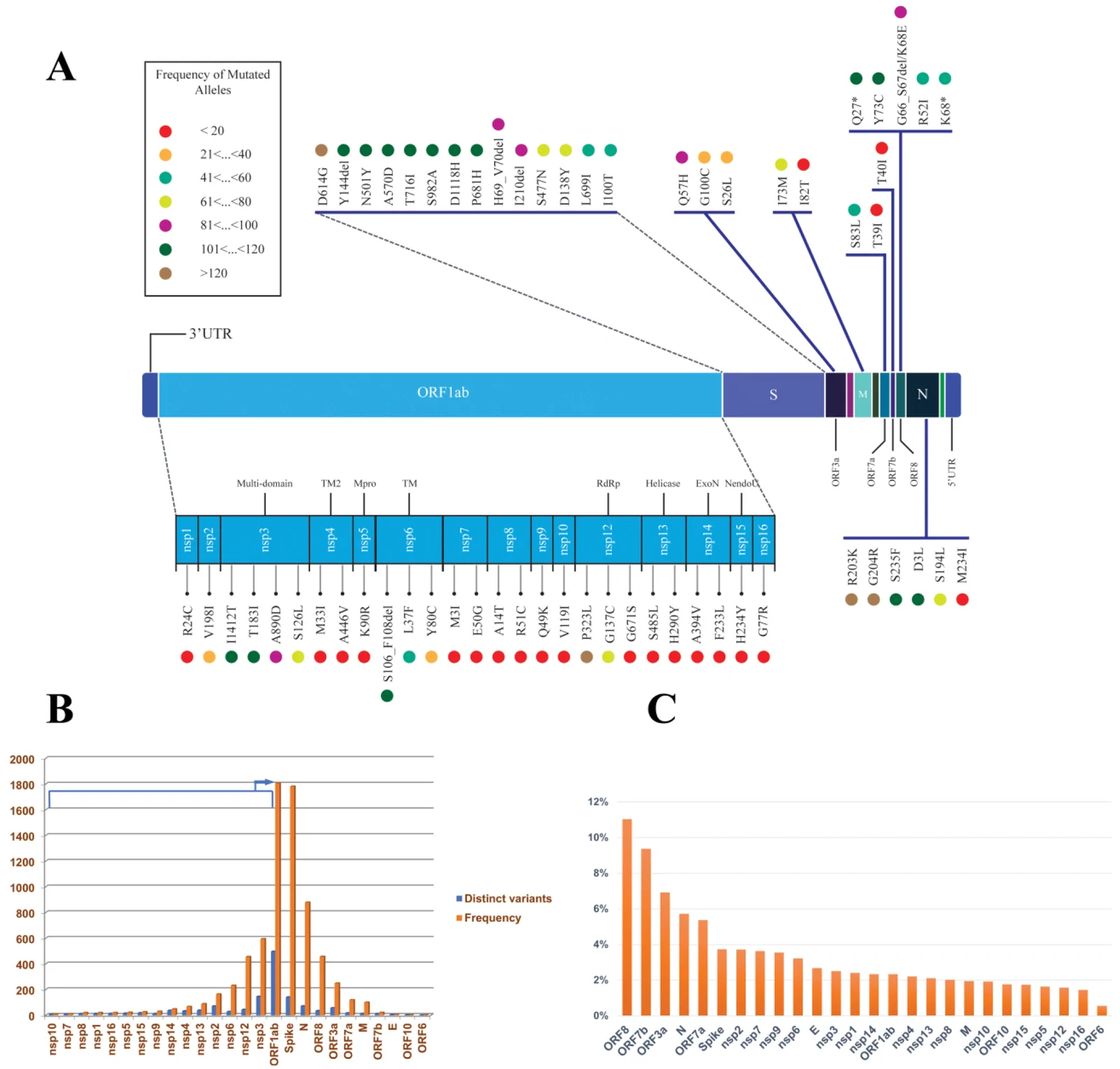
Mutational Profile of SARS-CoV-2 Viral Components in Iran; **(A)** shows a schematic representation of SARS-CoV-2 genes. The prominent mutations of each gene are drawn in colorful circles. The colors are based on the mutation frequencies indicated in the left panel; **(B)** shows the mutation rate of SARS-CoV-2 genes based on the number of distinct mutations (Blue) and frequency of the mutations (Orange) in each gene, **(C)** shows the adjusted mutation rate of each SARS-CoV-2 gene based on its length

 The majority of mutations occurred in the ORF1ab region (58%), as it covers 71% of the SARS-CoV-2 genome, followed by the spike (17%) and nucleocapsid (8%) genes which are the second and third longest genes (Figure 5B). However, by adjusting the distinct occurrence of mutations in each gene to its length (Figure 5C) the accurate mutation rate is higher in the accessory (ORF8, ORF7b, ORF3a, and ORF7a) and structural proteins such as the nucleocapsid and spike, rather than the non-structural genes such as nsp12 (RdRP).

 This result follows the previous understanding of ORF1ab being under-mutated compared to the other proteins, as the Nsp proteins, especially nsp12 (RdRP), have pivotal roles in RNA transcription and viral replication and should be more intact. While structural and accessory proteins such as ORF8 and N proteins are involved in virus-host interactions, the over-mutation adjusts the virus virulence or host immune avoidance.^25,35^ Consequently, the recurrent mutations of the following genes showed a frequency of less than 10% in our cohort (Red circles in Figure 5A); 11 Nsp genes (nsp1, nsp4, nsp5, nsp7, nsp8, nsp9, nsp10, nsp13, nsp14, nsp15, nsp16), one structural protein (E) and three accessory proteins (ORF6, ORF7b, and ORF10).

 The envelope (E) shows the lowest number of mutations with literally no recurrent mutation in our cohort among the structural proteins. This protein is involved in critical aspects of the viral life cycle, such as assembly, release, and virulence phases,^40,41^ and has shown the lowest mutation rate among the other SARS-CoV2 structural proteins.^25^

 Among the non-structural proteins, nsp3, nsp2, and nsp12 (RdRP) showed the most significant number of mutations, with percentages of 17%, 8%, and 5%, respectively (Figure 5B). The nsp3 protein is among the over-mutated SARS-CoV-2 proteins, but its C-terminal domain is significantly less mutated. Consistently, the recurrent nsp3 mutations in our cohort, nsp3:I1412T (6954T > C; ORF1a:I2230T), nsp3:T183I (3267C > T; ORF1a:T1001I), nsp3:A890D (5388C > A; ORF1a:A1708D), and nsp3:S126L (3096C > T; ORF1a:S944L) are outside this domain (Figures 5B and 5C).

 Furthermore, the nsp12 re-locates into the lowest limit of mutation rate by adjusting the number of mutations to its length (Figure 5C). The top nsp12 recurrent mutations, ORF1b:P314L (14408C > T; RdRp:P323L), ORF1b:G128C (13849G > T; RdRp:G137C) are located outside the RdRp-RNA interface, which is the most globally under-mutated region of this gene.^35^

###  Spike Mutations in SARS-CoV-2 Epidemic in Iran

 The spike protein (specifically its RBD) interacts directly with the ACE2 receptor on human cells and is therefore a preferred target for therapeutic antibodies and vaccines.^42^ Spike mutations significantly affect viral infectivity and dissemination of VOCs with global health impacts.^7^ At least 87% of RBD residues are mutated during the pandemic. However, most of these mutations are not persistent, except those developing VOCs.^43^

 In total, 141 spike mutations were detected in the SARS-CoV-2 epidemic in Iran, of which 90% occurred with less than 5% frequency (Table S3). The most frequent spike mutation was D614G, followed by 13 other frequent mutations based on the prominent lineages circulating in the country in different time intervals: B.1.1.7 (H69_V70del, Y144del, N501Y, A570D, P681H, T716I, S982A, D1118H in addition to I100T and L699I), B.1.1.413 (D138Y, S477N) and B.1.36 (I210del) (Figure 5A). Furthermore, 12 different RBD mutations were detected in our cohort (Table 3), of which 10 (83%) are represented with less than 5% frequency. All these mutations are previously detected around the world. Furthermore, seven of these mutations are the well-known RBD mutations flagged as mutations of concern and interest (E484K, N501Y, S477N, L452R, T478K, K417N, and E484Q). Interestingly, the S477N prevalence in Iran is higher than its cumulative prevalence globally, corresponding to the distinct B.1.1.413 lineage.

**Table 3:** Spike Mutations Located in the RBD Region

**Mutations**	**Prevalence/ Earliest Date in Iran**	**Type, Region**	**Prevalence/ Earliest Date in World**	**Corresponding Lineage in Iran**
N501Y	30%, 2020.10.14	Mutation of interest, RBM	37%, 2020.02.07	B.1.1.7 (98), B.1.1.7 + E484K (1), B.1.351 (2), B.1.36.31 (2), B.1.1 (2), B.1.1.413 (1)
S477N	22%, 2020.10.24	Mutation of interest, RBM	2%, 2020.01.28	B.1.1.413 (68), B.1.1.7 (6), B.1.1.99 (1), B.1.260 (2), B.1.36.10 (1), B.1.533 (1)
L452R	4.5%, 2021.01.19	Mutation of interest, RBM	32%, 2020.03.03	B.1.617.2(13), B.1.617.1 (2), B.1.36 (1)
T478K	4%, 2021.04.00	Mutation of interest (?), RBM	30%, 2020.03.15	B.1.617.2(13)
E484K	0.8%, 2021.04.03	Mutation of concern, RBM	6%, 2020.02.01	B.1.351(2), B.1.1.7 + E484K (1)
K417N	0.6%, 2021.04.03	Mutation of interest, RBD	1%, 2020.02.15	B.1.351(2)
E484Q	0.6%, 2021.04.00	Mutation of interest (?), RBM	< 0.5%, 2020.03.03	B.1.617.1(2)
F490S	0.6%, 2021.01.00	RBM	< 0.5%, 2020.04.05	B.1.1.7 (1), B.1.36 (1)
A344S	0.3%, 2020.04.13	RBD	< 0.5%, 2020.03.07	B.4 (1)
Y365C	0.3%, 2021.01.00	RBD	< 0.5%, 2020.12.29	B.1.1.7 (1)
P479L	0.3%, 2021.04.29	RBM	< 0.5%, 2020.04.03	B.1.617.2 (1)
V483A	0.3%, 2021.01.00	RBM	< 0.5%, 2020.02.29	B.1 (1)

 Overall, the spike mutation content of the SARS-CoV-2 epidemic in Iran is similar to other parts of the world, and no country-specific mutation with a significant rise in the epidemic was detected except the significant rise in the combination of [D138Y-S477N-D614G] mutations.

## Discussion

 Today, over one year after the declaration of the COVID-19 pandemic, genome surveillance projects are frequently performed in all countries to track the spread of SARS-CoV-2 variants.^38^ The current study reports the complete genome sequencing of 369 SARS-CoV-2 viral isolates of the Iranian outbreak from March 2020 until May 2021. Therefore, this study does not cover the fifth and sixth disease waves in Iran, which are dominated by Delta (B.1.617.2) and Omicron (B.1.1.529) variants, respectively (unpublished data).

 This study provides a comprehensive picture of the temporal and geographical dynamics of the main SARS-CoV-2 clades/lineages circulating in Iran. It introduces the 19A clade (B.4 lineage) dominating the first disease wave (Spring 2020),^15^ followed by 20A (B.1.36), 20B (B.1.1.413), and 20I (B.1.1.7), dominating the second (Summer 2020), third (Autumn 2020) and forth (Spring 2021) disease waves, respectively. It also shows that the COVID-19 patients in winter 2021 were mainly exposed and infected by a mixture of circulating 20A (B.1.36), 20B (B.1.1.413), and 20I (B.1.1.7) clades, competing in a diminishing manner for 20A/20B, paralleled with a growing rise of 20I (B.1.1.7), eventually prompting the fourth outbreak peak in spring 2021. Furthermore, our study provides supporting information about the entry of the Delta variant (21A clade) in April 2021, which gave rise to the fifth disease wave in summer 2021. This study shows that almost all the previous circulating SARS-CoV-2 lineages disappeared with the rise of the Alpha variant, which itself might have been replaced upon the entry of the Delta variant in April.

 The temporal dynamic is comparable to the global perspective. The pandemic was dominated by the 19A/19B clades until March 2020 and replaced by the 20A clade (appearance of D614G mutation) until mid-September. However, we never detected any viral isolates from the 19B clade (Pango A lineages) in Iran, except for one sample from the A23.1 lineage (19B clade) in December 2020. The 19B clade did not rise in Iran, while in February 2021, its resurgence was reported in some countries (dominated by the A.23.1, A.27 and, A.2.5 lineages).^44^ From mid-September 2020 onward, the pandemic was dominated by the 20E clade, while we detected 20B dominance, which is more similar to the pandemic in Asia, in which 20B was the leading clade in January.^38^

 Although coronaviruses show higher replication fidelity, thousands of spontaneous mutations have accumulated in the SARS-CoV-2 genome since its emergence in 2019, and 79% of its amino acids are mutated at least once.^35^ Mutation investigation in SARS-CoV-2 genomes provides reliable information about the epidemic status, as these mutations may affect the viral transmission rate and the host’s antiviral immune response.^45^ Therefore, the simultaneous mutation surveillance of SARS-CoV-2 genomes in this time interval nicely reflects the disease epidemic status in Iran.

 The prominence of nsp6:L37F mutation in the first disease wave may partly explain the unrecognized transmission of the disease at the start of the epidemic in Iran. This missense mutation has shown a significant correlation to asymptomatic SARS-CoV-2 infections. It is unfavorable for viral transmission, possibly compromising the virus’s ability to confront innate cellular defense.^46^ However, we should not overlook the misdiagnosed patients or the limited testing capacities at the epidemic’s beginning.^15,47^

 The appearance of the second outbreak peak can be justified by a new mutational trend in the country (S:D614G, RdRp:P323L, and ORF3a:Q57H). The renowned D614G mutation is located outside the Receptor Binding Domain (RBD) of the spike protein but possibly increases viral transmission. Additionally, P323L encodes a more error-prone RdRp, correlating with increased viral genetic diversity, allowing the virus to spread more effectively in different environmental conditions and populations.^48^ On the other hand, Q57H causes a dramatic change in protein structure, affecting the binding affinity of Orf3a–S and Orf3a–Orf8 protein interactions, and finally may be correlated with enhanced viral virulence,^48,49^ as the ORF3a is an essential protein for viral cytotoxicity and activates the inflammasome.

 Later, the accumulation of R203K/G204R, S477N, and D138Y on the mutational burden of the epidemic explains the surge of the third disease wave in autumn 2020. The adjacent nucleotide substitutions, 28881G > A, 28882G > A, and 28883G > C, designated the B.1.1 lineage and arose on the background of D614G mutation through homologous recombination. These mutations encode the adjacent R203K/G204R missense mutations in the nucleocapsid serine/arginine‐rich (SR‐rich) linker region,^37,45^ which is involved in modulating its multimerization, and cellular localization. Therefore, R203K/G204R mutations may destabilize the protein, followed by a decrease in the overall structural flexibility, and can finally increase the virulence of SARS-CoV-2.^45,50-52^ Finally, the predominance of B.1.1.7 defining mutations justifies the observation of the fourth disease wave in the country.

 Our study highlights three hallmarks of the SARS-CoV-2 outbreak in Iran. First, the B.4 lineage mediates the primary phase. Second, a notable rise in the B.1.1 lineage carrying a combination of [D138Y-S477N-D614G] spike mutations in autumn 2020 and winter 2021, recently assigned as B.1.1.413. Although this lineage is present in other regions such as Turkey and Europe, Iran seems to be the only country that experienced a disease wave due to the growing prevalence of this lineage at that time. Third, the B.1.1.7 outbreak mediated by two types of B.1.1.7 sequences, with 46.5% prevalence of [S:I100T-S: L699I + B.1.1.7 defining mutations] in Iran compared to < 0.5% in the world. However, this mutational combination did not show a higher transmission rate.

 In conclusion, the sequences obtained through this study provided a good resource for the monthly estimation of the SARS-CoV-2 mutational burden in the country. Continuous monitoring of SARS-CoV-2 mutations is a requisite, as it facilitates tracking the possible formation of VOCs in each region. Although some new mutations and distinct lineages were observed in the Iranian outbreak, no specific SARS-CoV-2 variant was introduced in the course of this study in Iran. This information can also be used to refine the primers and probes applied in RT-PCR-based diagnosis, as the accumulating nucleotide variations can disturb primer binding sites, leading to a high percentage of false-negative test results of even up to 50% in some reports.^53^ The RdRp and nucleocapsid genes are among the targets of widely-used commercial kits in diagnostic laboratories in Iran. Our data displayed a high frequency of mutations in the nucleocapsid gene. It also revealed some recurrent mutations in RdRp with a fixed high frequency, such as P323L, G137C, and G671S. Therefore, the sequences obtained through this study could be applied for refining and updating primers and eventually increasing the country’s quality of diagnostic tests.

## Supplementary Materials


Supplementary file 1 contains Figures S1-S6.

Supplementary file 2 contains Table S1.

Supplementary file 3 contains Table S2

Supplementary file 4 contains Table S3.
