# Supplementary material for: Disease Waves of SARS-CoV-2 in Iran Closely Mirror Global Pandemic Trends
Source: Arch Iran Med. 2022 Aug 1;25(8):508–22. doi: 10.34172/aim.2022.83 (PMC13661075; doi:10.34172/aim.2022.83)

Arch Iran Med. August 2022;25(8):508-522
10.34172/aim.2022.83

Disease Waves of SARS-CoV-2 in Iran Closely Mirror Global Pandemic Trends

Zohreh Fattahi, PhD[1,2#]; Marzieh Mohseni, PhD[1,2#]; Maryam Beheshtian, MD, PhD[1,2]; Ali Jafarpour, MSc[3,4]; Khadijeh Jalalvand, BSc[1]; Fatemeh Keshavarzi, MSc[1]; Hanieh Behravan, BSc[1]; Fatemeh Ghodratpour, MSc[1]; Farzane Zare Ashrafi, MSc[1]; Marzieh Kalhor, MSc[1]; Maryam Azad, BSc[2]; Mahdieh Koshki, BSc[2]; Azam Ghaziasadi, PhD[3]; Mohamad Soveyzi, MSc[1]; Alireza Abdollahi, MD, PhD[5]; Seyed Jalal Kiani, PhD[6]; Angila Ataei-Pirkooh, PhD[6]; Iman Rezaeiazhar, MSc[3]; Farah Bokharaei-Salim, PhD[6]; Mohammad Reza Haghshenas, PhD[7]; Farhang Babamahmoodi, MD, PhD[7]; Zakiye Mokhames, PhD[8]; Alireza Soleimani, MD, PhD[9]; Zohreh Elahi, MSc[1]; Masood Ziaee, MD, PhD[10]; Davod Javanmard, PhD[10]; Shokouh Ghafari, PhD[10]; Akram Ezani, MSc[11]; Alireza Ansari Moghaddam, PhD[12]; Fariba Shahraki-Sanavi, PhD[12]; Seyed Mohammad Hashemi Shahri, MD, PhD[13]; Azarakhsh Azaran, PhD[14]; Farid Yousefi, MD, PhD[15]; Afagh Moattari, PhD[16]; Mohsen Moghadami, MD, PhD[17]; Hamed Fakhim, PhD[18]; Behrooz Ataei, MD, PhD[18]; Elahe Nasri, MD, PhD[18]; Vahdat Poortahmasebi, PhD[19]; Mojtaba Varshochi, MD, PhD[20]; Ali Mojtahedi, PhD[21]; Farid Jalilian, PhD[22]; Mohammad Khazeni, MSc[23]; Abdolvahab Moradi, PhD[24]; Alijan Tabarraei, PhD[24]; Ahmad Piroozmand, PhD[25]; Yousef Yahyapour, PhD[26]; Masoumeh Bayani, MD, PhD[26]; Fatemeh Tavangar, BSc[27]; Mahmood Yaghoubi, PhD[28]; Fariba Keramat, MD, PhD[29]; Mahsa Tavakoli, PhD[30]; Tahmineh Jalali, PhD[30,31]; Mohammad Hassan Pouriayevali, PhD[30,31]; Mostafa Salehi-Vaziri, PhD[30,31]; Hamid Reza Khorram Khorshid, MD, PhD[1]; Reza Najafipour, MD, PhD[1]; Reza Malekzadeh, MD[32]; Kimia Kahrizi, MD[1]; Seyed Mohammad Jazayeri, MD, PhD[3]; Hossein Najmabadi, PhD[1,2*]

Full list of authors' affiliations is available at the end of the article.

Supplementary file 1

Figure S1. Detailed overview of lineages in each month and each outbreak peak.

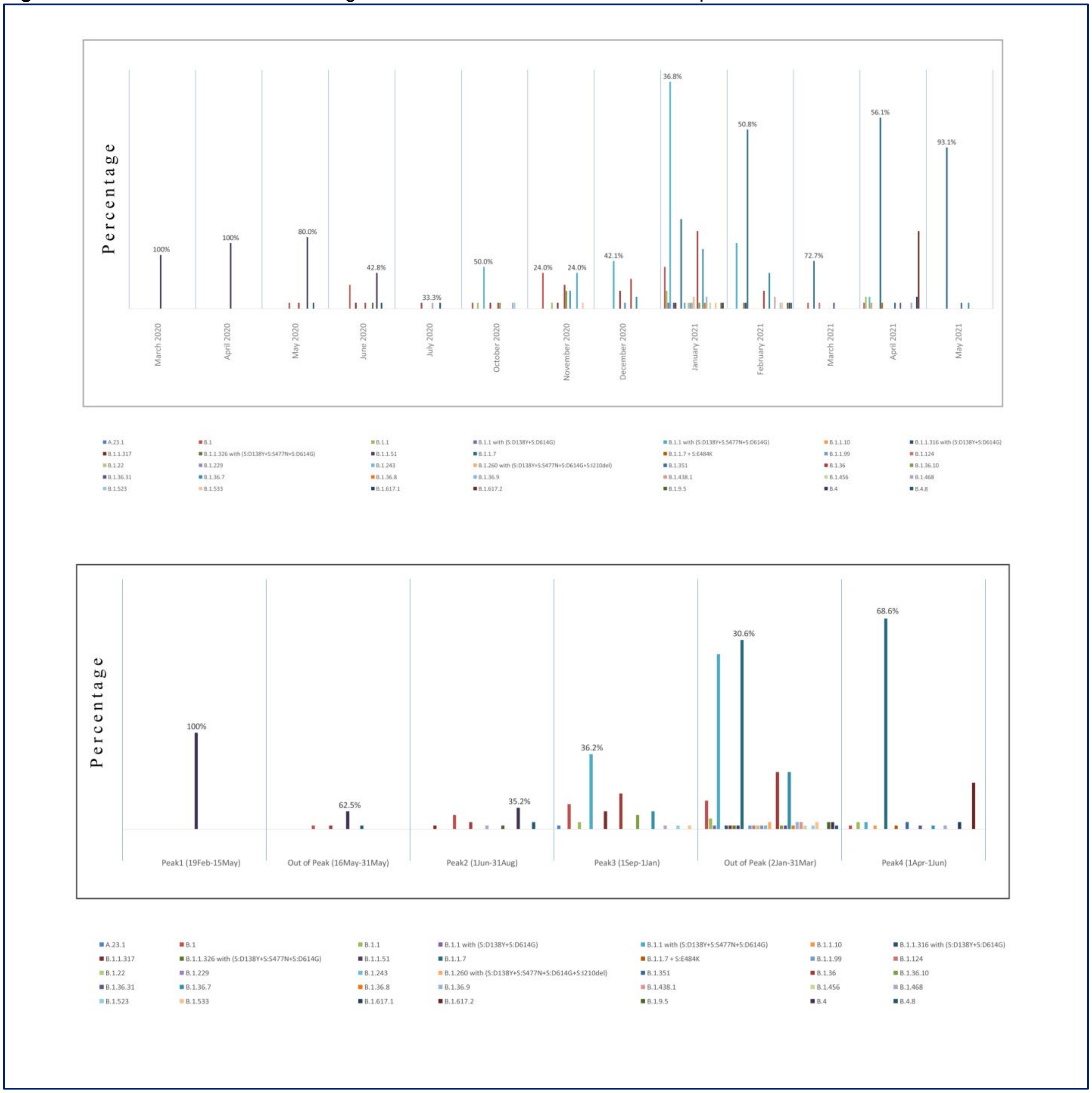

Figure S2. Top mutations in the first wave of SARS-CoV-2 infection (March-May 2020)

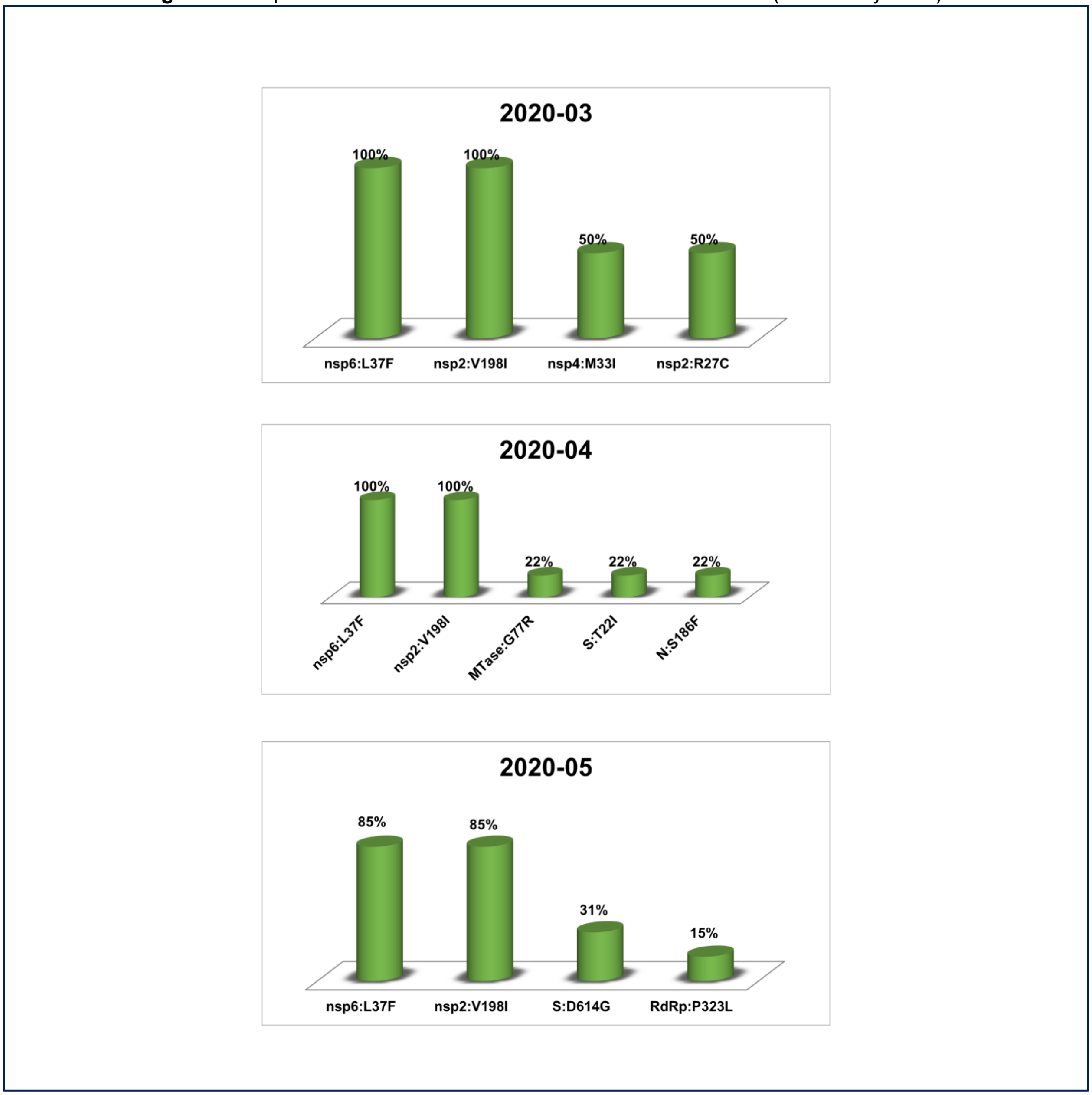

Figure S3. Top mutations in the second wave of SARS-CoV-2 infection (June-August 2020)

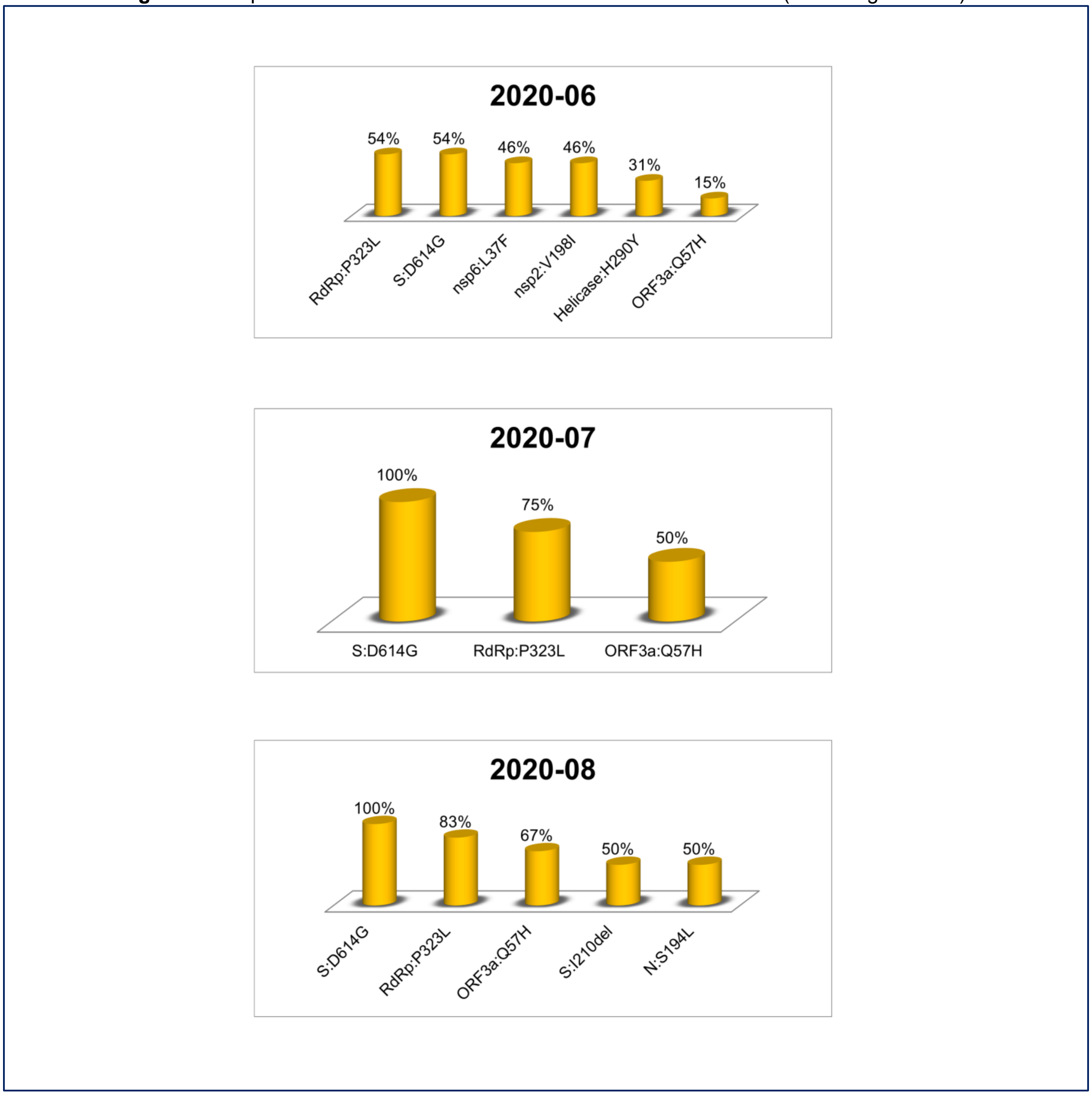

Figure S4. Top mutations in the third wave of SARS-CoV-2 infection (September-December 2020)

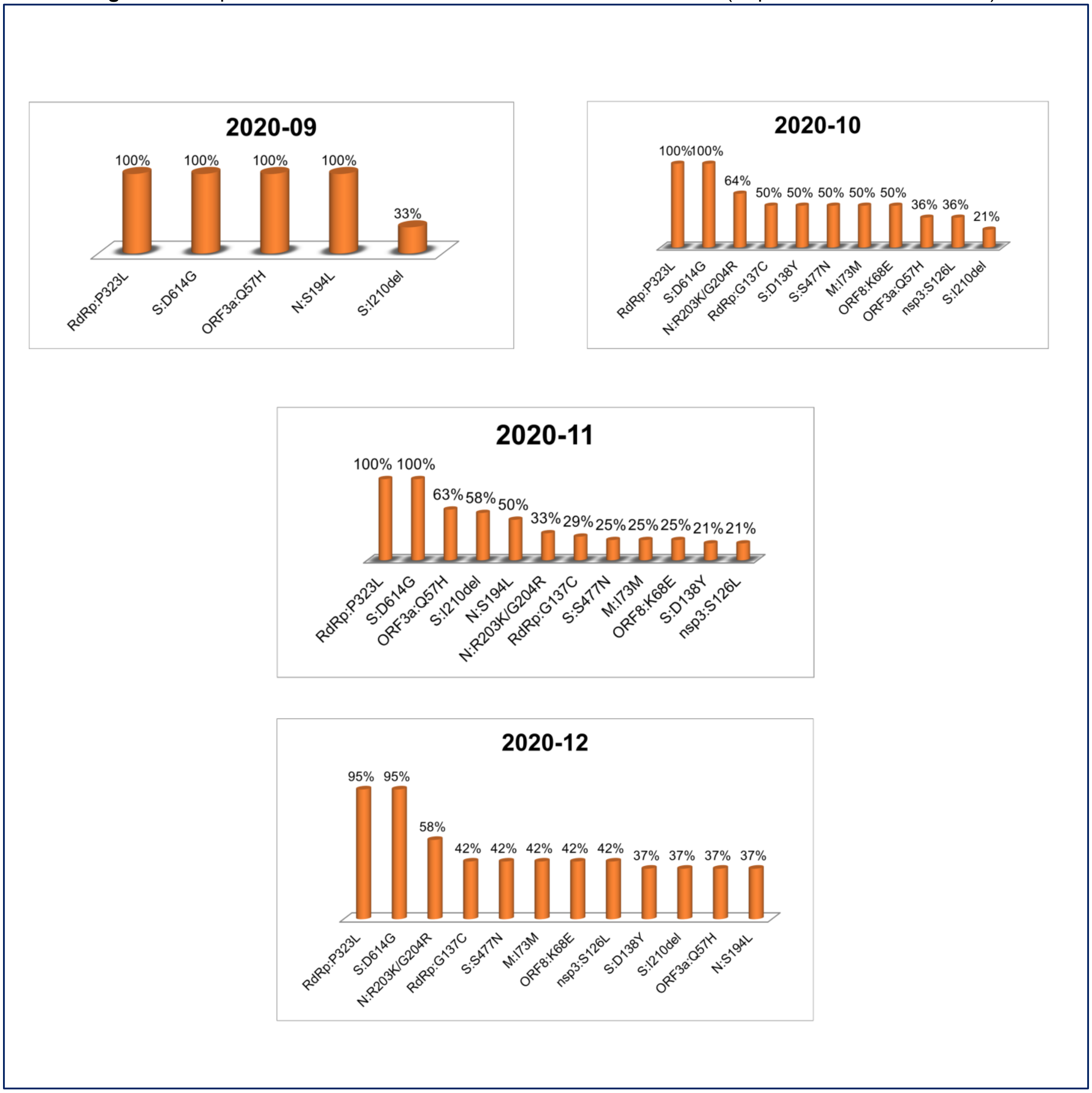

Figure S5. Top mutations in January-March 2021

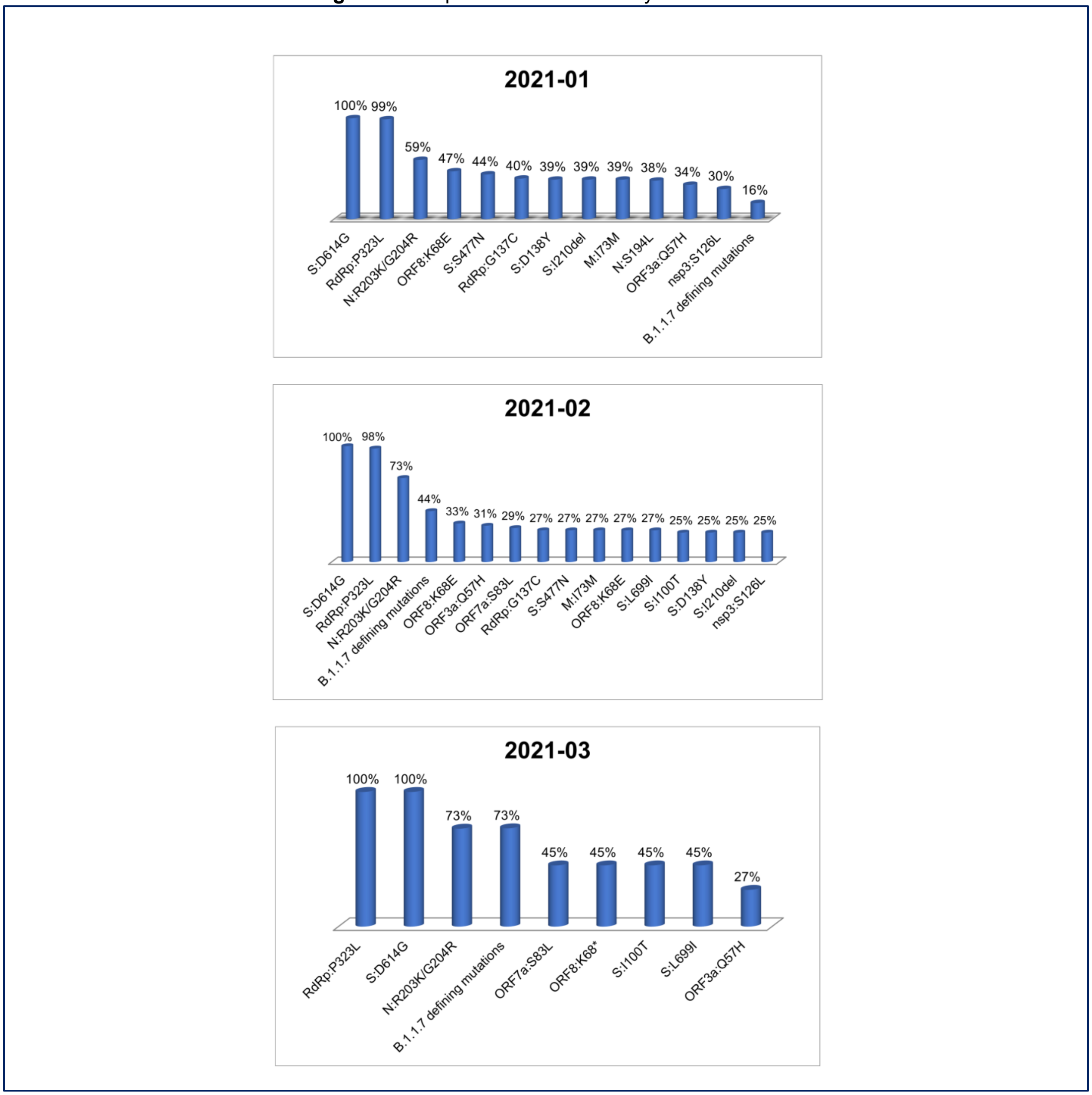

Figure S6. Top mutations in the fourth wave of SARS-CoV-2 infection (April-May 2021)

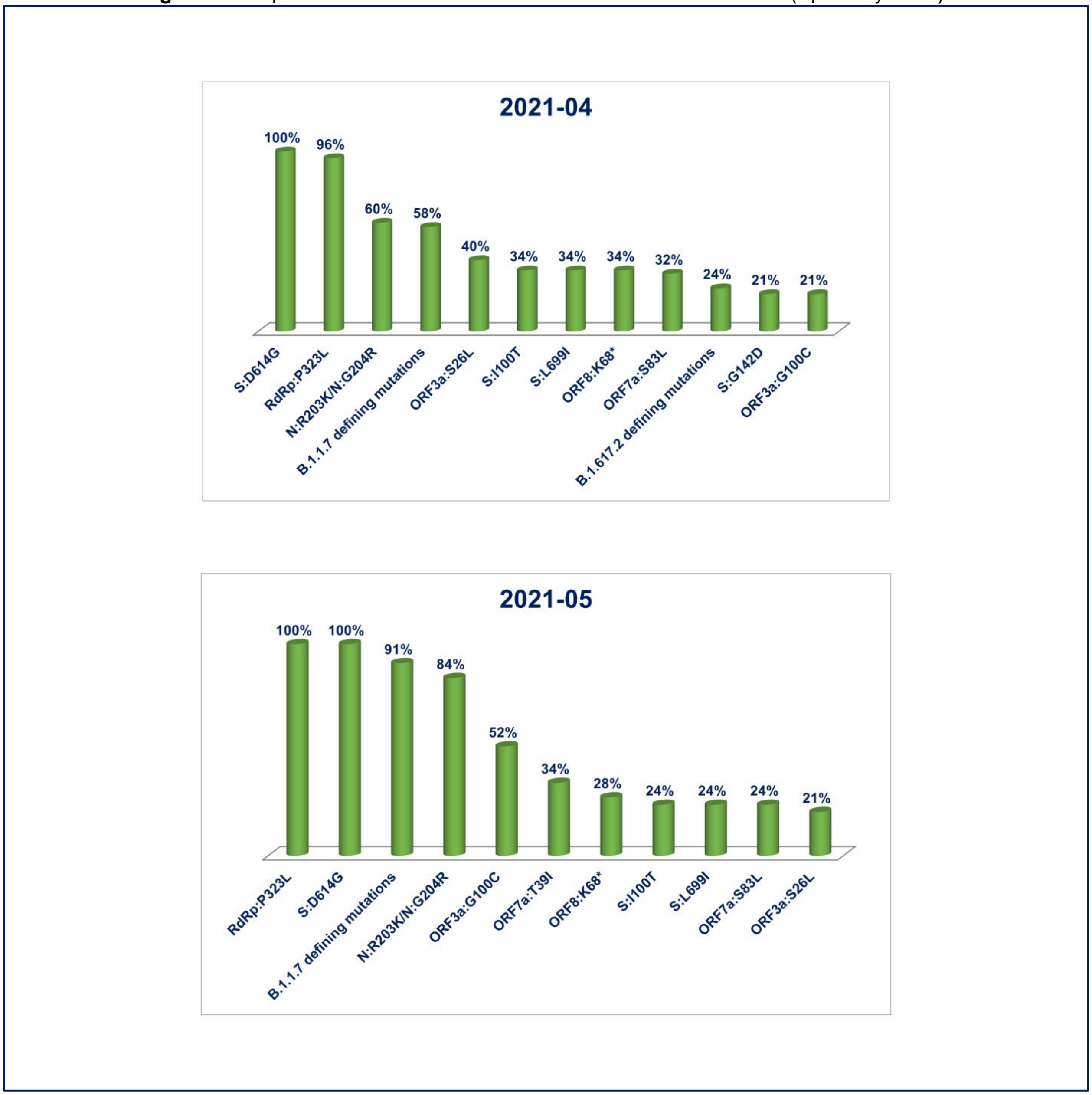

Supplement: Supplementary file 1 [file aim-25-508-s001.pdf]
